# Social Media and Internet Driven Study Recruitment: Evaluating a New Model for Promoting Collaborator Engagement and Participation

**DOI:** 10.1371/journal.pone.0118899

**Published:** 2015-03-16

**Authors:** Chetan Khatri, Stephen J. Chapman, James Glasbey, Michael Kelly, Dmitri Nepogodiev, Aneel Bhangu, J. Edward Fitzgerald, on behalf of the STARSurg Committee

**Affiliations:** 1 Imperial College London Medical School, London, United Kingdom; 2 University of Leeds, Leeds, United Kingdom; 3 Cardiff University Medical School, Cardiff, United Kingdom; 4 University of Liverpool Medical School, Liverpool, United Kingdom; 5 Norwich Academic Foundation Programme, Norwich, United Kingdom; 6 Academic Department of Surgery, 4^th^ Floor, Old Queen Elizabeth Hospital, University of Birmingham, College of Medical and Dental Sciences, West Midlands, B15 2TH, Birmingham, United Kingdom; 7 University College London, London, United Kingdom; Rollins School of Public Health, Emory University, UNITED STATES

## Abstract

**Aims:**

A substantial challenge facing multicentre audit and research projects is timely recruitment of collaborators and their study centres. Cost-effective strategies are required and fee-free social media has previously been identified as a potential conduit. We investigated and evaluated the effectiveness of a novel multi-format social media and Internet strategy for targeted recruitment to a national multicentre cohort study.

**Methods:**

Interventions involved a new Twitter account, including weekly live question-and-answer sessions, a new Facebook group page, online YouTube presentations and an information page on a national association website. Link tracking analysis was undertaken using Google Analytics, which was then related to subsequent registration. Social influence was calculated using the proprietary Klout score.

**Results:**

Internet traffic analysis identified a total of 1562 unique registration site views, of which 285 originated from social media (18.2%). Some 528 unique registrations were received, with 96 via social media platforms (18.2%). Traffic source analysis identified a separate national association webpage as resulting in the majority of registration page views (15.8%), followed by Facebook (11.9%), Twitter (4.8%) and YouTube (1.5%). A combination of publicity through Facebook, Twitter and the dedicated national association webpage contributed to the greatest rise in registration traffic and accounted for 312 (48%) of the total registrations within a 2-week period. A Twitter ‘social influence’ (Klout) score of 42/100 was obtained during this period.

**Conclusions:**

Targeted social media substantially aided study dissemination and collaborator recruitment. It acted as an adjunct to traditional methods, accounting for 18.2% of collaborator registration in a short time period with no associated financial costs. We provide a practical model for designing future recruitment campaigns, and recommend Facebook, Twitter and targeted websites as the most effective adjuncts for maximising cost-effective study recruitment.

## Introduction

Timely participant or collaborator recruitment is a major challenge in launching a large, multi-centre study. One eight-year review noted that only 31% of multi-centre randomised controlled trials achieved their original recruitment targets in a timely manner [1]. Non-completed studies can delay the introduction of new healthcare interventions or increase the time in which the population is exposed to a suboptimal treatment [2]. Delays in recruitment may also increase financial costs [3], which risks biasing investigators and funders towards smaller more rapidly delivered studies instead of longer term projects.

Engaging with clinicians or patients to stimulate participant recruitment can be difficult. Clinician involvement can be hindered by issues such as time limitations, loss of professional autonomy, concern for patients, or a perceived lack of rewards and recognition [4,5]. Patients may be worried about the inconvenience, costs, or uncertainty of treatment, with some having a particular preference to receiving (or not receiving) the treatment [4,5]. These factors can be compounded in both groups by a lack of awareness to study opportunities. While patient recruitment has been the topic of extensive previous study, the area of clinician engagement is less well understood.

Previous studies have proposed social media as a potential new modality for study promotion and recruitment [2,6–8] and have highlighted the reduced costs associated with this novel approach. With Facebook and Twitter having over one billion users each and YouTube having over 645 million users [9–11] many individuals access such platforms on a daily basis. Studies investigating social media uptake specifically within the medical profession have reported usage rates of between 13–47% amongst doctors [12] with 48% of all users logging on to use Facebook on any given day [10]. This has made social media a potentially valuable resource for promoting studies and collaborator recruitment, which could lead to more efficient patient recruitment.

Social media is largely a free-to-use medium, which can be harnessed to advertise and recruit medical professionals for academic projects. It can potentially reach large, targeted populations rapidly, providing recruitment opportunities to interested but unknown individuals who may not otherwise be engaged within standard professional or institutional contact networks. Although existing studies have described the use of social media to recruit patients, there is a paucity of literature detailing its role in recruiting medical collaborators, and minimal guidance on how to practically undertake this.

The STudent Audit and Research in Surgery (STARSurg) group was formed based on a previously described collaborative research model [13]. The group established a novel network of medical students, junior doctors, and supervising surgeons, to facilitate data collection for a national, multi-centre cohort study.

This paper reports and evaluates a multi-modal social media and Internet strategy to promote and recruit collaborators for a national, multi-centre study. In addition, based on our experiences, we provide a practical model for future studies, for others who may wish to utilise this novel approach.

## Methods

### Research Question

This study investigated the use of social media and online strategies as a cost-effective method of collaborator recruitment for a national cohort study, and evaluated the relative outcomes of these. We hypothesised that the use of these online platforms could potentially increase recruitment over traditional methods with no associated financial outlay. We designed a novel Internet and social media collaborator recruitment model to test this.

### Ethics

According to regulations set out by the Health Resource Authority decision tool (http://www.hra-decisiontools.org.uk/research/), this evaluation of collaborator recruitment did not require ethical approval. The national cohort study protocol was reviewed by the National Research Ethics Service and a Research Ethics Committee, that the anonymous, observational nature of data collection meant that this did not require formal research ethics registration.

### STARSurg Collaborative Model

The STudent Audit and Research in Surgery (STARSurg) research and audit collaborative network (http://www.starsurg.org/) undertook a national, multicentre cohort study using a protocol-driven, medical student investigator-led approach to determine the safety profile of non-steroidal anti-inflammatory drugs (NSAIDs) following gastrointestinal (GI) surgery [14–16].

Collaborators were responsible for gaining local audit approval and undertaking prospective data collection. A structured hierarchy of collaborator support was provided, which included local clinical faculty, geographical regional coordinators and a dedicated steering committee.

### Subjects and Settings

Junior doctors and medical students were targeted and invited to act as collaborators. An online registration via a dedicated Google form (http://www.google.com/google-d-s/createforms.html) was utilised for collaborator registration. A single web link to this registration form was created and used universally across all promotional interventions to facilitate link tracking and analytics.

### Recruitment strategies

Five interventions were used to recruit collaborators. All social media interventions were used exclusively for study-related promotion, and were accessible on the Internet free of charge without the need for registration or subscription fees to access.

1. Targeted emails to medical student unions, university surgical and academic societies and professional bodies.
2. A dedicated information webpage on the Association of Surgeons in Training (ASiT) website (http://www.asit.org/news/STARSurgUK1)
3. A dedicated Twitter feed targeting student unions, surgical and academic societies and postgraduate professional bodies (https://twitter.com/STARSurgUK).
4. A dedicated Facebook group page, linked to the Twitter feed, which could inform potential participants of recent developments (https://www.facebook.com/STARSurgUK).
5. A dedicated YouTube channel, including an introductory video with the purpose of explaining the project and protocol to potential collaborators (https://www.youtube.com/channel/UCDewmFYVD1wfciYEwy8UHtQ?feature=watch).

### Twitter

A dedicated Twitter account (Twitter, Inc., San Francisco, USA) was created at the start of the seven-week recruitment period. Tweets are short messages, which are limited to 140 characters, and can include links to external websites or images. Regular Tweets encouraged potential collaborators to join our study with a specific web link leading to the registration page included in the message. We asked groups to ‘retweet’ (forward) these messages to their own followers and continued this throughout the recruitment period. Strategies to disseminate the study included targeting specific organisations with medical student and junior doctor interests. These groups included: student unions, student medical, surgical and academia societies, selected individuals and professional postgraduate bodies. These organisations were ‘followed’ leading to an organic growth of reciprocal ‘followers’ that could see our posts.

### Facebook

A dedicated Facebook group page (Facebook, Inc., California, USA) was created at the start of the recruitment period and linked to the Twitter feed, ensuring the same content was delivered through both social media platforms. Facebook pages can be ‘liked’ by users who wish to follow the content delivered through the group.

Additional informative posts were added to other Facebook groups’ pages that were deemed to be of interest where members of the groups/pages would be able to see our post. Posts consisted of a short paragraph encouraging students and junior doctors to collaborate and included the same link to the registration page. For larger, professional organisations, private messages were sent to request they forward on our advert, which included a link to the online registration page.

### YouTube

A dedicated YouTube channel (YouTube, LLC, California, USA) was created at the start of the recruitment period with a single 7:53 minute video uploaded. This consisted of a narrated, step-by-step presentation, which aimed to explain the project and protocol to potential collaborators. To encourage participation, a link to the registration form was placed in the comments section.

### Data Analysis

Descriptive analysis was performed using Google Analytics (http://www.google.com/analytics), an integral feature of Google shortened links. Social media influence was calculated using the ‘Klout Score’. Klout (Klout Inc, San Francisco, US) is a free online social media analytics tool used to rank users according to their online social influence by means of their proprietary Klout Score. This is a numerical value between 1 and 100, measured using access- and analysis-related metrics from linked social media accounts in the preceding 90 days. These may include follower counts, retweets and the influence of the users who retweet your own messages. The average Klout score across all social media users is 40 [17]. Financial currency conversions are based on prevailing market rates on 29^th^ November 2014 using the UK Forex exchange rate, rounded to the nearest whole unit of currency.

## Results

### Collaborator Demographics

A total of 476 collaborators collected data at 109 hospital centres across the UK. Analysis of the collaborator registration page showed a total of 1562 unique views, which resulted in 527 registrations to give a 33.7% (527/1562) conversion rate.

### Comparative Recruitment Between Different Platforms

At the end of the registration period, using traffic source analysis from Google Analytics, the dedicated study webpage hosted on the national association website resulted in the greatest interest, accounting for 15.7% (245/1562) of views on the collaborator registration page, and generating 83 registrations.

Within the social media category, the Facebook group page attracted 160 ‘likes’ after 378 posts. This formed the majority of social media traffic, accounting for 11.9% views (186/1562) and 63 registrations. The Twitter page gained 236 followers from 378 tweets, resulting in 4.87% of views (76/1562) and resulting in 25 collaborator registrations. One video was published on our YouTube channel, which attracted 372 views. The link from this YouTube video page accounted for 1.47% (23/1562) of views to our registration page with 8 subsequent registrations.

Traditional methods including emailing and organic spread accounted for the majority of collaborator registration site views, with 66.1% of views (1032/1562) eventually leading to 348 registrations.

### Outcomes and Impact

**Fig 1:**
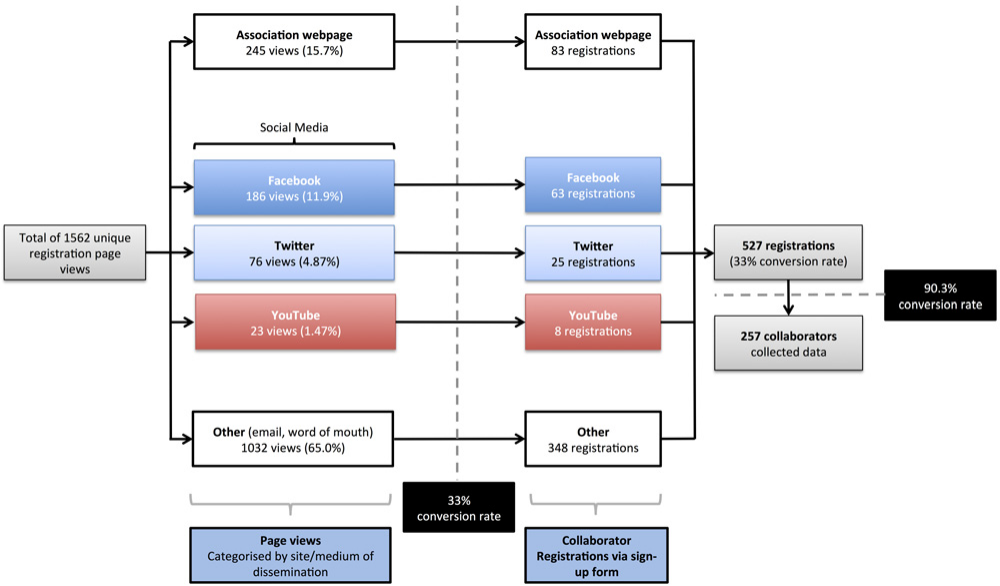
Detailed comparative breakdown of collaborator recruitment by social media and standard methods.

A flowchart detailing the comparative study recruitment through these different approaches is provided in Fig. 1. A Twitter impact ‘social influence’ (Klout) score of 42/100 was calculated during this seven-week time period, indicating an above average social media influence gained over the seven-week period.

## Discussion

This study indicates that targeted use of free social media and internet resources substantially assisted study dissemination in a cost-effective manner, accounting for 18.2% of collaborator recruitment in a short time period. This resulted in a substantially higher click-through rate (33.7%) to recruit collaborators when compared to other studies recruiting members of the general public [8,18,19]. We have demonstrated that effective targeting of groups of interest can result in a high level of recruitment with no additional costs. This strategy is however time intensive and requires effective identification of groups of interest, together with approaches that harness and maximise the potential benefits of multi-modal social media usage.

The broad use of social media for non-medical marketing has developed rapidly in recent years; however medical and scientific communities have been slower to adapt this new technology for their own needs. The potential advantages and disadvantages of using this new approach for study recruitment are summarised in Table 1.

**Table 1:** Advantages and disadvantages of using targeted social media to aid study recruitment.

Advantages	Disadvantages
Free to use other than the time spent establishing and maintaining a social media presence	May introduce demographic selection bias by recruiting a younger, more internet-active population
Easy and intuitive to use	Requires working knowledge of relevant, active interest groups and influencers
Rapid dissemination of information	Overall potentially lower recruitment conversion rates compared to traditional methods
Can be a useful adjunct to existing traditional models of study recruitment	Requires time commitment to develop content and update this
Used by up to 80.8% of medical students [30]	May not be as effective for recruitment of established medical professionals (compared to recruitment of medical students) due to lower usage and penetration [12]; this is however increasing with time
Potentially allows recruitment to extend across international borders	Biased against countries or regions with restricted access to social media
Facilitates two-way communication and effective engagement with the target population	Social media platforms can be access-restricted by hospital IT departments, which may impede the flow of communication.
Provides an accessible medium by which to distribute electronic study resources including media, electronic protocols and information documents	May bias against those internationally who lack internet or electronic resources to access these
Offers the potential to engage collaborators who may not otherwise participate via traditional methods	

The current model of free social media use by the wider population is largely based on paid-for advertising by businesses seeking promotion. There have been previous reports of paid-for Facebook advertising to assist with study recruitment, with Kapp *et al*. reporting the use of adverts available to the general population [8]. Their promotion was ‘reached’ by 373,225 women of whom 250 clicked the advert. Out of those who clicked the advert, only 9 proceeded past the introduction page leading to a click through rate of 3.2%. Paid Facebook advertising to recruit patients to randomised controlled trials (RCTs) has been reported to cost between €Euros 12 ($USD 15.28) and $USD 20.14 (€Euros 15.81) per recruited participant [6,18]. Our study found a click-through rate of 33.7% without paid-for advertising, which is likely due to the highly targeted nature of our social media activities compared to general advertising. As such, although our readership was smaller, those viewing it may have been more likely to engage and follow through to registration. Paid advertising may still be beneficial, as it also generates an increase in the associated Facebook or Twitter page visits, which can potentially enhance future recruitment and aid organic growth and awareness. Compared with alternative methods of trial recruitment, paid advertising via Facebook has been shown to be more cost-effective, with conventional methods costing between $USD 20 (€Euros 15.71) and $USD 500 (€Euros 392.63) [20,21].

Traditional methods such as face-to-face recruitment, which in our study consisted of lectures and talks at universities and hospitals, are also time intensive. This problem is further compounded when centres are located nationally or internationally. Bhatnager *et al*. described that whilst personally meeting with study sites increased recruitment by a modest mean of one patient per month, this effect was only limited for 120 days, prompting suggestion that further interventions would be required to sustain this model [22]. However, it is notable that when specifically qualified and trained personal were used for site visits, site recruitment increased significantly, albeit with a greater cost and time intensity associated with this method [23].

Other methods for recruiting patients, like random digit phone calling, can result in both high staff time consumption as well as high recruitment costs per participant, potentially reaching up to $USD 1075 (€Euros 844.16) per patient [24]. Recruitment via social media, especially free-of-charge activities rather than paid adverts, potentially represents a cheaper, more time-effective method of recruitment. Once the appropriate social media accounts have been established and target groups identified, on-going promotion incurs little time-cost. These can also be re-purposed in future for other related studies and activities, maximising the return on the initial time investment required to set these up.

The usefulness of Facebook and Twitter are not limited to paid adverts, although varying success has been previously reported by the use of free services in social media. Whilst a study by Godino *et al*. found that 12.2% of participants were recruited from social media [25], others have found social media to be the most successful method of recruiting participants to a study [26]. The relative success or otherwise reported to date is likely to be dictated by numerous factors including a mix of the strategies used on these, the particular target audience and the nature of the study being recruited to. If carefully considered, a focused marketing strategy may allow Twitter to be used as the sole platform to collect data [27].

A novel feature in this study was an online presentation uploaded to YouTube. Researchers often struggle to provide the same high-quality message to all participants [19]. This method is far less time intensive than face-to-face interactions as well providing a standardised message. For those engaged by the protocol, a link placed at the bottom of this video helped enhance recruitment and in this study lead to an extra 23 clicks to our registration page, translating to 8 eventual collaborators.

Depending on the nature of the study, social media may however introduce a bias into recruited participants. Previous studies have noted that an online advertisement paradigm resulted in recruitment of a younger population sample [28,29], which cannot necessarily be generalised to the rest of the population. In this study the target audience (study collaborators) fitted with the younger demographic profile of social media users, enhancing recruitment. However, social media may not be as effective when targeting more senior groups of clinicians. Whilst 80.8% of medical students within England use social media [12,30], this rate drops to much lower rates of between 12.8% to 46.7% within established medical professionals [12]. In addition, social media has little or no impact on the cohort of professionals who do not use it. This may imply that those wishing to recruit professionals rather than medical students or younger junior doctors as potential collaborators may experience lower rates of engagement.

An additional general weakness of this recruitment method is that it introduces selection bias by potentially over-representing those individuals who spend the most amount of time on social media communities such as Facebook and Twitter. However, this bias is similar to many other forms of population sampling conducted over the Internet. The time spent on Facebook by an individual has been shown to be positively correlated to two personality traits: neuroticism and total loneliness, whilst a negative correlation was shown to be present for time spent and conscientiousness [31]. While unlikely to impact on our national cohort study, the implications of these observations will need to be taken into account by others depending on the nature of the study.

Although this study recruited a total of 528 investigators, only 476 ultimately collected data. This dropout rate is likely due to numerous factors. Firstly, there was often an oversubscription within many universities, with the number of collaborators wanting to collect data outnumbering the possible sites for data collection. Secondly there was a small cohort who entered their contact details incorrectly and therefore was lost to further contact. Lastly, there were collaborators who expressed an interest in collecting data, but later withdrew due to other commitments. Such dropout rates need to be considered when setting target numbers for recruitment. Unfortunately the data does not enable us to link those ultimately collecting data with their mode of initial engagement (social media, website, or email), and these confounding factors influencing the dropout means that such linkage would not add insight. However, in future studies it would be interesting to investigate whether the mode of initial engagement influenced the likelihood of eventual participation.

Although this study was limited to national participation, the ease with which social media reaches across borders potentially provides particularly cost-effective methods for recruitment to international studies. However, targeting this may be more difficult in countries where the influencing groups and individuals are less well known, and may also be biased towards countries with adequate and unrestricted use of social media. Additionally, this study relates to targeting healthcare professionals and medical students; the degree to which these findings can be extrapolated to direct patient recruitment for studies is unknown.

Future corroborating research in this area is needed to investigate the overall effectiveness of social media on the recruitment of medical professionals including a comparison of different strategies and approaches to facilitate this. Within this, it is important that future research should specifically determine variation of productivity and reliability of collaborators recruited by social media versus recruited via other methods. Finally, there is a need for an increased awareness of the usefulness of social media amongst the wider scientific community as a potential conduit to disseminate study protocols and to recruit collaborators.

### Proposed Practical Model for Social Media Driven Collaborator Recruitment

**Fig 2:**
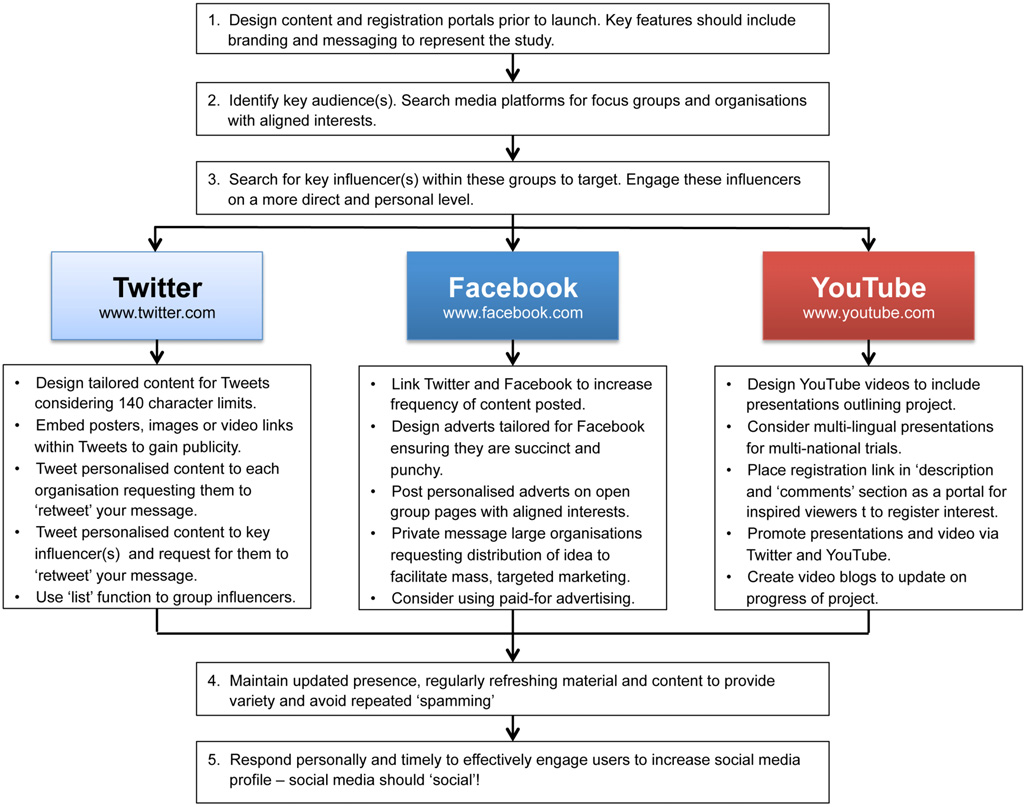
Suggested practical model recruitment strategies for utilising social media.

Based on our experiences using targeted social media for collaborator recruitment, we have developed a proposed practical model for developing future approaches to this (Fig. 2). Our model outlines the practical steps that need to be considered and put in place in order to construct such a targeted campaign and how to go about deploying this. In particular, care should be taken in selecting groups to be targeted, as irrelevant groups will lead to ineffective uptake.

## Conclusions

Targeted social media substantially aided study dissemination and collaborator recruitment as an adjunct to traditional methods, accounting for 18.2% of collaborator registration in a short time period with no associated financial outlay. We propose a practical model for designing future recruitment campaigns and recommend Facebook, Twitter and targeted websites as the most effective approach to achieving this, this novel method of communication can supplement more traditional methods, maximising rapid study recruitment in a cost-effective manner.
